# 5-(Pyridinium-3-yl)tetra­zol-1-ide hexa­aqua­magnesium dichloride

**DOI:** 10.1107/S160053681005419X

**Published:** 2011-01-15

**Authors:** Jing Dai, Xin-Yuan Chen

**Affiliations:** a Ordered Matter Science Research Center, College of Chemistry and Chemical Engineering, Southeast University, Nanjing 210096, People’s Republic of China

## Abstract

In the title compound, (C_6_H_5_N_5_)_2_[Mg(H_2_O)_6_]Cl_2_, the asymmetric unit contains one zwitterionic 5-(pyridinium-3-yl)tetra­zol-1-ide mol­ecule, one half of an [Mg(H_2_O)_6_]^2+^ cation (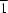 symmetry) and one chloride ion. The Mg^II^ ion is surrounded by six water mol­ecules, with their O atoms located at the apices, exhibiting a slightly distorted octa­hedral coordination. Mg—O bond lengths range from 2.0526 (14) to 2.0965 (16) Å [mean value = 2.068 Å]. The pyridine and tetra­zole rings are nearly coplanar and only twisted from each other by a dihedral angle of 5.68 (1)°. The zwitterionic organic mol­ecules, anions and cations are connected by O—H⋯Cl, O—H⋯N and N—H⋯Cl hydrogen bonds, leading to the formation of a three-dimensional network.

## Related literature

For tetra­zole derivatives, see: Zhao *et al.* (2008 ▶); Fu *et al.* (2008 ▶, 2009 ▶). For the crystal structures and properties of related compounds, see: Fu *et al.* (2007 ▶, 2009 ▶); Fu & Xiong (2008 ▶).
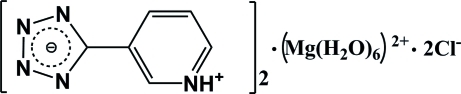


## Experimental

### 

#### Crystal data

(C_6_H_5_N_5_)_2_[Mg(H_2_O)_6_]Cl_2_
*M* *_r_* = 497.61
Triclinic, 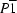

*a* = 7.4354 (15) Å
*b* = 8.4232 (17) Å
*c* = 9.5817 (19) Å
α = 94.06 (3)°
β = 90.71 (3)°
γ = 110.67 (3)°
*V* = 559.60 (19) Å^3^
*Z* = 1
Mo *K*α radiation
μ = 0.37 mm^−1^
*T* = 298 K
0.40 × 0.05 × 0.05 mm

#### Data collection

Rigaku SCXmini diffractometer
Absorption correction: multi-scan (*CrystalClear*; Rigaku, 2005 ▶) *T*_min_ = 0.89, *T*_max_ = 0.95
5836 measured reflections
2552 independent reflections
2086 reflections with *I* > 2σ(*I*)
*R*_int_ = 0.029

#### Refinement

*R*[*F* ^2^ > 2σ(*F* ^2^)] = 0.041
*wR*(*F* ^2^) = 0.101
*S* = 1.09
2552 reflections
142 parameters
6 restraints
H-atom parameters constrained
Δρ_max_ = 0.27 e Å^−3^
Δρ_min_ = −0.25 e Å^−3^

### 

Data collection: *CrystalClear* (Rigaku, 2005 ▶); cell refinement: *CrystalClear*; data reduction: *CrystalClear*; program(s) used to solve structure: *SHELXTL* (Sheldrick, 2008 ▶); program(s) used to refine structure: *SHELXTL*; molecular graphics: *SHELXTL*; software used to prepare material for publication: *SHELXTL*.

## Supplementary Material

Crystal structure: contains datablocks I, global. DOI: 10.1107/S160053681005419X/bx2339sup1.cif

Structure factors: contains datablocks I. DOI: 10.1107/S160053681005419X/bx2339Isup2.hkl

Additional supplementary materials:  crystallographic information; 3D view; checkCIF report

## Figures and Tables

**Table 1:** Hydrogen-bond geometry (Å, °)

*D*—H⋯*A*	*D*—H	H⋯*A*	*D*⋯*A*	*D*—H⋯*A*
O1*W*—H1*WA*⋯N4^i^	0.85	1.90	2.737 (2)	169
O1*W*—H1*WB*⋯Cl1^ii^	0.85	2.34	3.1848 (17)	174
O2*W*—H2*WA*⋯N5^iii^	0.85	1.94	2.775 (2)	167
O2*W*—H2*WB*⋯Cl1^iv^	0.85	2.46	3.2764 (19)	163
N1—H1*A*⋯Cl1^iv^	0.86	2.25	3.088 (2)	165
O3*W*—H3*WA*⋯N2	0.85	1.89	2.735 (2)	177
O3*W*—H3*WB*⋯Cl1	0.85	2.34	3.1822 (17)	172

Symmetry codes: (i) 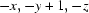; (ii) 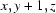; (iii) 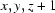; (iv) 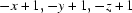.

**Fig. 1.**
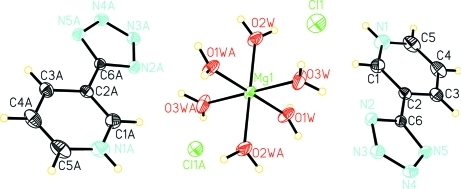
A view of the title compound with the atomic numbering scheme. Displacement ellipsoids were drawn at the 30% probability level.

**Fig. 2.**
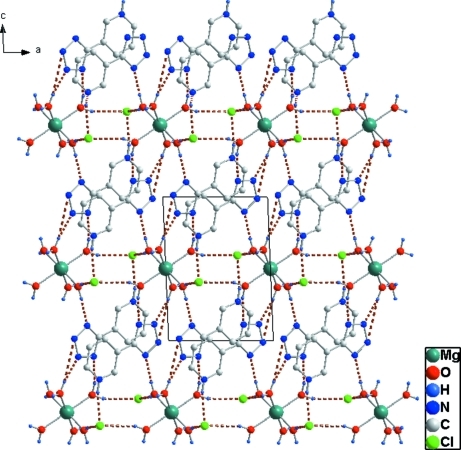
The crystal packing of the title compound, showing the three-dimensional hydrogen-bonded network. H atoms not involved in hydrogen bonding (dashed line) have been omitted for clarity.

## supplementary crystallographic information

### Comment

Tetrazole compounds have attracted more attention as phase transition dielectric materials for its application in micro-electronics, memory storage. With the purpose of obtaining phase transition crystals of 3-(1*H*-tetrazol-5-yl)pyridine compounds, its interaction with various metal ions has been studied and a series of new materials have been elaborated with this organic molecule (Zhao *et al.*, 2008; Fu *et al.*, 2008; Fu *et al.*, 2007; Fu & Xiong 2008). In this paper, we describe the crystal structure of the title compound, 3-(pyridinium-5-yl)tetrazol-1-ide hexaaquamagnesium(II) dichloride.

In the title compound, (C_6_H_5_N_5_)_2_.[Mg(H_2_O)_6_]^2+^.2Cl, the asymmetric unit consists of one zwitterionic 3-(pyridinium-5-yl)tetrazol-1-ide molecule, one half of an [Mg(H_2_O)_6_]^2+ ^cation ( 1 symmetry) and one chloride ion. The magnesium(II) ion is surrounded by six water molecules with their O atoms located at the apices exhibiting a slightly distorted octahedral coordination. Mg—O bond distances range from 2.0526 (14) to 2.0965 (16)Å (mean value 2.0681 (15)Å). In the zwitterionic organic molecules, the pyridine and tetrazole rings are nearly coplanar and only twisted from each other by a dihedral angle of 5.68 (1)°. The geometric parameters of the tetrazole rings are comparable to those in related molecules (Zhao *et al.*, 2008; Fu *et al.*, 2009).

In crystal structure, the complex cations [Mg(H_2_O)_6_]^2+^ and Cl^-^ anions are linked through O–H···Cl H-bonds into broad infinite cation-anion sheet parallel to the (0 0 1) plane. The two-dimensional sheets are linked by organic molecules through O—H···N and N—H···Cl H-bonds into a three-dimensional framework (Table 1 and Fig.2).

### Experimental

MgCl_2_.6H_2_O (2 mmol) and 3-(1*H*-tetrazol-5-yl)pyridine (2 mmol, 0.528 g) were dissolved in 70% methanol aqueous solution, and them 2 ml HBr was added. Single crystals suitable for X-ray diffraction analysis were obtained from slow evaporation of the solution at room temperature after two weeks.

### Refinement

All H atoms attached to C and N atoms were fixed geometrically and treated as riding with C–H = 0.93 Å (aromatic) and N–H = 0.86 Å with *U*_iso_(H) = 1.2*U*_eq_(C or N). All aqueous hydrogen atoms were calculated geometrically, O–H = 0.85 Å and were refined using a riding model and *U*_iso_(H) = 1.5*U*_eq_(O).

### Crystal data

**Table:**

(C_6_H_5_N_5_)_2_[Mg(H_2_O)_6_]Cl_2_	*Z* = 1
*M**_r_* = 497.61	*F*(000) = 258
Triclinic, *P*1	*D*_x_ = 1.477 Mg m^−^^3^
Hall symbol: -P 1	Mo *K*α radiation, λ = 0.71073 Å
*a* = 7.4354 (15) Å	Cell parameters from 2552 reflections
*b* = 8.4232 (17) Å	θ = 3.2–27.5°
*c* = 9.5817 (19) Å	µ = 0.37 mm^−^^1^
α = 94.06 (3)°	*T* = 298 K
β = 90.71 (3)°	Block, colourless
γ = 110.67 (3)°	0.40 × 0.05 × 0.05 mm
*V* = 559.60 (19) Å^3^	

### Data collection

**Table:**

Rigaku SCXmini diffractometer	2552 independent reflections
Radiation source: fine-focus sealed tube	2086 reflections with *I* > 2σ(*I*)
graphite	*R*_int_ = 0.029
Detector resolution: 13.6612 pixels mm^-1^	θ_max_ = 27.5°, θ_min_ = 3.2°
CCD_Profile_fitting scans	*h* = −9→9
Absorption correction: multi-scan (*CrystalClear*; Rigaku, 2005)	*k* = −10→10
*T*_min_ = 0.89, *T*_max_ = 0.95	*l* = −12→12
5836 measured reflections	

### Refinement

**Table:**

Refinement on *F*^2^	Primary atom site location: structure-invariant direct methods
Least-squares matrix: full	Secondary atom site location: difference Fourier map
*R*[*F*^2^ > 2σ(*F*^2^)] = 0.041	Hydrogen site location: inferred from neighbouring sites
*wR*(*F*^2^) = 0.101	H-atom parameters constrained
*S* = 1.09	*w* = 1/[σ^2^(*F*_o_^2^) + (0.0398*P*)^2^ + 0.1577*P*] where *P* = (*F*_o_^2^ + 2*F*_c_^2^)/3
2552 reflections	(Δ/σ)_max_ < 0.001
142 parameters	Δρ_max_ = 0.27 e Å^−^^3^
6 restraints	Δρ_min_ = −0.25 e Å^−^^3^

### Special details

**Table:**

Geometry. All e.s.d.'s (except the e.s.d. in the dihedral angle between two l.s. planes) are estimated using the full covariance matrix. The cell e.s.d.'s are taken into account individually in the estimation of e.s.d.'s in distances, angles and torsion angles; correlations between e.s.d.'s in cell parameters are only used when they are defined by crystal symmetry. An approximate (isotropic) treatment of cell e.s.d.'s is used for estimating e.s.d.'s involving l.s. planes.
Refinement. Refinement of *F*^2^ against ALL reflections. The weighted *R*-factor *wR* and goodness of fit *S* are based on *F*^2^, conventional *R*-factors *R* are based on *F*, with *F* set to zero for negative *F*^2^. The threshold expression of *F*^2^ > σ(*F*^2^) is used only for calculating *R*-factors(gt) *etc*. and is not relevant to the choice of reflections for refinement. *R*-factors based on *F*^2^ are statistically about twice as large as those based on *F*, and *R*- factors based on ALL data will be even larger.

### Fractional atomic coordinates and isotropic or equivalent isotropic displacement parameters (Å^2^)

**Table:**

	*x*	*y*	*z*	*U*_iso_*/*U*_eq_	
Mg1	0.0000	0.5000	0.5000	0.0313 (2)	
O1W	0.0599 (2)	0.69394 (16)	0.36976 (13)	0.0459 (4)	
H1WA	0.0113	0.6775	0.2869	0.069*	
H1WB	0.1228	0.7998	0.3859	0.069*	
O2W	0.2487 (2)	0.64382 (19)	0.62087 (14)	0.0528 (4)	
H2WA	0.2766	0.6238	0.7025	0.079*	
H2WB	0.3531	0.7163	0.5961	0.079*	
N2	0.2278 (2)	0.46353 (18)	0.10243 (15)	0.0317 (3)	
C6	0.3333 (2)	0.5845 (2)	0.02382 (17)	0.0263 (3)	
C2	0.4917 (2)	0.7372 (2)	0.07879 (18)	0.0299 (4)	
N3	0.1006 (2)	0.34796 (18)	0.01273 (16)	0.0357 (3)	
N4	0.1310 (2)	0.39758 (19)	−0.11389 (16)	0.0362 (4)	
N5	0.2776 (2)	0.54786 (18)	−0.11105 (15)	0.0336 (3)	
O3W	0.1562 (2)	0.3929 (2)	0.37477 (15)	0.0565 (4)	
H3WA	0.1824	0.4149	0.2906	0.085*	
H3WB	0.1934	0.3113	0.3914	0.085*	
C3	0.6097 (3)	0.8510 (2)	−0.0093 (2)	0.0370 (4)	
H3	0.5879	0.8315	−0.1058	0.044*	
C1	0.5289 (3)	0.7704 (2)	0.2209 (2)	0.0416 (5)	
H1	0.4520	0.6969	0.2821	0.050*	
N1	0.6760 (3)	0.9086 (2)	0.2710 (2)	0.0528 (5)	
H1A	0.6978	0.9268	0.3602	0.063*	
C4	0.7593 (3)	0.9931 (2)	0.0469 (3)	0.0490 (5)	
H4	0.8379	1.0697	−0.0117	0.059*	
C5	0.7908 (3)	1.0201 (3)	0.1877 (3)	0.0543 (6)	
H5	0.8912	1.1151	0.2264	0.065*	
Cl1	0.31120 (8)	0.08959 (6)	0.40654 (6)	0.05028 (17)	

### Atomic displacement parameters (Å^2^)

**Table:**

	*U*^11^	*U*^22^	*U*^33^	*U*^12^	*U*^13^	*U*^23^
Mg1	0.0380 (5)	0.0285 (4)	0.0220 (4)	0.0051 (3)	0.0002 (3)	0.0023 (3)
O1W	0.0647 (9)	0.0284 (7)	0.0290 (7)	−0.0025 (6)	−0.0112 (6)	0.0042 (5)
O2W	0.0463 (8)	0.0564 (9)	0.0343 (7)	−0.0092 (7)	−0.0087 (6)	0.0112 (6)
N2	0.0320 (7)	0.0299 (7)	0.0284 (7)	0.0051 (6)	0.0011 (6)	0.0025 (6)
C6	0.0244 (8)	0.0257 (8)	0.0266 (8)	0.0066 (6)	0.0011 (6)	0.0009 (6)
C2	0.0260 (8)	0.0260 (8)	0.0358 (9)	0.0079 (7)	−0.0005 (7)	−0.0019 (7)
N3	0.0304 (8)	0.0296 (8)	0.0405 (9)	0.0030 (6)	−0.0007 (6)	0.0004 (6)
N4	0.0322 (8)	0.0347 (8)	0.0363 (8)	0.0065 (6)	−0.0073 (6)	−0.0022 (6)
N5	0.0328 (8)	0.0335 (8)	0.0285 (8)	0.0043 (6)	−0.0026 (6)	0.0032 (6)
O3W	0.0863 (12)	0.0675 (10)	0.0347 (8)	0.0479 (9)	0.0192 (8)	0.0157 (7)
C3	0.0323 (9)	0.0317 (9)	0.0450 (11)	0.0082 (8)	0.0038 (8)	0.0052 (8)
C1	0.0380 (10)	0.0383 (10)	0.0391 (10)	0.0037 (8)	−0.0024 (8)	−0.0062 (8)
N1	0.0490 (10)	0.0494 (11)	0.0477 (10)	0.0071 (8)	−0.0102 (8)	−0.0201 (8)
C4	0.0358 (10)	0.0284 (10)	0.0754 (16)	0.0023 (8)	0.0065 (10)	0.0041 (9)
C5	0.0372 (11)	0.0329 (11)	0.0798 (17)	0.0004 (9)	−0.0046 (11)	−0.0158 (10)
Cl1	0.0557 (3)	0.0341 (3)	0.0536 (3)	0.0067 (2)	−0.0008 (2)	0.0045 (2)

### Geometric parameters (Å, °)

**Table:**

Mg1—O1W	2.0526 (14)	C2—C3	1.393 (3)
Mg1—O1W^i^	2.0526 (14)	N3—N4	1.308 (2)
Mg1—O3W^i^	2.0552 (15)	N4—N5	1.346 (2)
Mg1—O3W	2.0552 (15)	O3W—H3WA	0.8500
Mg1—O2W	2.0965 (16)	O3W—H3WB	0.8499
Mg1—O2W^i^	2.0965 (16)	C3—C4	1.383 (3)
O1W—H1WA	0.8500	C3—H3	0.9300
O1W—H1WB	0.8499	C1—N1	1.338 (2)
O2W—H2WA	0.8500	C1—H1	0.9300
O2W—H2WB	0.8499	N1—C5	1.344 (3)
N2—C6	1.334 (2)	N1—H1A	0.8600
N2—N3	1.339 (2)	C4—C5	1.356 (3)
C6—N5	1.333 (2)	C4—H4	0.9300
C6—C2	1.462 (2)	C5—H5	0.9300
C2—C1	1.375 (3)		
			
O1W—Mg1—O1W^i^	180.00 (5)	C1—C2—C3	118.19 (17)
O1W—Mg1—O3W^i^	91.30 (6)	C1—C2—C6	120.00 (16)
O1W^i^—Mg1—O3W^i^	88.70 (6)	C3—C2—C6	121.80 (16)
O1W—Mg1—O3W	88.70 (6)	N4—N3—N2	109.00 (14)
O1W^i^—Mg1—O3W	91.30 (6)	N3—N4—N5	110.09 (14)
O3W^i^—Mg1—O3W	180.000 (1)	C6—N5—N4	104.18 (14)
O1W—Mg1—O2W	88.73 (6)	Mg1—O3W—H3WA	124.6
O1W^i^—Mg1—O2W	91.27 (6)	Mg1—O3W—H3WB	127.9
O3W^i^—Mg1—O2W	89.27 (7)	H3WA—O3W—H3WB	107.1
O3W—Mg1—O2W	90.73 (7)	C4—C3—C2	120.02 (19)
O1W—Mg1—O2W^i^	91.27 (6)	C4—C3—H3	120.0
O1W^i^—Mg1—O2W^i^	88.73 (6)	C2—C3—H3	120.0
O3W^i^—Mg1—O2W^i^	90.73 (7)	N1—C1—C2	120.00 (19)
O3W—Mg1—O2W^i^	89.27 (7)	N1—C1—H1	120.0
O2W—Mg1—O2W^i^	180.0	C2—C1—H1	120.0
Mg1—O1W—H1WA	121.5	C1—N1—C5	122.59 (19)
Mg1—O1W—H1WB	130.3	C1—N1—H1A	118.7
H1WA—O1W—H1WB	107.9	C5—N1—H1A	118.7
Mg1—O2W—H2WA	125.4	C5—C4—C3	119.6 (2)
Mg1—O2W—H2WB	129.5	C5—C4—H4	120.2
H2WA—O2W—H2WB	103.6	C3—C4—H4	120.2
C6—N2—N3	105.15 (14)	N1—C5—C4	119.60 (18)
N5—C6—N2	111.57 (14)	N1—C5—H5	120.2
N5—C6—C2	124.30 (15)	C4—C5—H5	120.2
N2—C6—C2	124.13 (15)		

Symmetry codes: (i) −*x*, −*y*+1, −*z*+1.

### Hydrogen-bond geometry (Å, °)

**Table:**

*D*—H···*A*	*D*—H	H···*A*	*D*···*A*	*D*—H···*A*
O1W—H1WA···N4^ii^	0.85	1.90	2.737 (2)	169
O1W—H1WB···Cl1^iii^	0.85	2.34	3.1848 (17)	174
O2W—H2WA···N5^iv^	0.85	1.94	2.775 (2)	167
O2W—H2WB···Cl1^v^	0.85	2.46	3.2764 (19)	163
N1—H1A···Cl1^v^	0.86	2.25	3.088 (2)	165
O3W—H3WA···N2	0.85	1.89	2.735 (2)	177
O3W—H3WB···Cl1	0.85	2.34	3.1822 (17)	172

Symmetry codes: (ii) −*x*, −*y*+1, −*z*; (iii) *x*, *y*+1, *z*; (iv) *x*, *y*, *z*+1; (v) −*x*+1, −*y*+1, −*z*+1.
